# Atherosclerosis: An Integrative East-West Medicine Perspective

**DOI:** 10.1155/2012/148413

**Published:** 2012-04-05

**Authors:** Hao Xu, Dazhuo Shi, Keji Chen

**Affiliations:** Cardiovascular Diseases Center, Xiyuan Hospital, China Academy of Chinese Medical Sciences, Beijing 100091, China

## Abstract

Recent understanding of atherosclerosis and coronary heart disease has shifted the focus from lumen stenosis to vulnerable plaque, from lipid deposit to inflammatory reaction, and from vulnerable plaque to vulnerable patient. This has led to a new direction of treatment consisting of intervening the inflammatory reaction, stabilizing the vulnerable plaque, inhibiting thrombosis after plaque rupture, and treating the vulnerable patient instead of treating lumen stenosis. This seems to mirror the traditional Chinese medicine (TCM) focus on prevention and on the vulnerable patient with treatment matched to the pattern dysfunction and dysregulation using the Chinese herbal medicine multitargeted approach. Given the convergence of both the East and the West conceptualization of atherosclerosis, it is hopeful that the integrative East-West approach will facilitate early detection and more effective treatment of the vulnerable patients with coronary heart disease.

## 1. Introduction

Atherosclerosis (AS) is the most common type of arteriosclerosis. It mainly involves the large and middle muscular arteries, especially aorta, coronary and cerebral arteries, which often leads to serious outcomes such as sudden cardiac death, unstable angina pectoris, acute myocardial infarction, stroke, and intermittent claudication due to vessel obliteration or plaque rupture and subsequent thrombosis. In the beginning of the 21st century, we are facing serious challenges of cardiovascular disease (CVD). Although it is becoming less lethal, CVD prevalence is incessantly increasing, and it is still the most common cause of death. How to prevent AS and reduce the incidence and mortality of CVD have been one of the most important health-related issues all the time.

However, biomedicine is at its limits nowadays when confronting degenerative diseases, stress-related diseases, and most chronic diseases. It lacks reference to the self-healing capacity of the human mind and body and focuses on parts rather than the whole, treatment rather than prevention, the suffering disease rather than the diseased person. Confronted with these problems, more and more far-sighted Western scholars began to lay their eyes on traditional Chinese medicine (TCM) [1–3]. Drugs with Chinese herbal medicines as raw materials are increasingly favored by people all over the world for their unique advantages in preventing and curing diseases, rehabilitation, and health care. The benefit of TCM in CVD was also demonstrated in several multicenter clinical trials in recent years [4–7]. More importantly, the unique theory of TCM might also have some implications for the renewal of thinking in fighting against CVD [8]. Therefore, we reviewed traditional understanding and shifted concepts on AS pathophysiology along the track of previous studies and read these transitions taking full advantage of TCM theory together with our experimental and clinical studies in recent years, so as to provide an integrative East-West medicine perspective for future AS prevention and treatment.

## 2. Updated Concept of Atherosclerosis

### 2.1. From Emphasizing “Luminal Stenosis” to Highlighting “Vulnerable Plaques”

With the deep understanding and active control of AS risk factors, dramatic advances have been made in primary prevention of chronic cardiovascular diseases since 1990s. However, there is still lack of effective measure to prevent acute cardiovascular events (ACEs), which cause 20 million deaths worldwide per year. Most of the victims die suddenly without any prior symptoms.

The previous studies focused on the severity of coronary stenosis, taking coronary heart disease (CHD) as an example of AS, and highlighted detection of severe luminal stenosis and subsequent treatment of percutaneous coronary intervention (PCI). The development or improvement of coronary stenosis is also regarded as an important indicator to evaluate the state of illness or therapeutic effect. However, angiographic studies on patients before myocardial infarction showed that the majority of subsequent events involved sites with less than 70% obstruction. It indicated that the severity of stenosis was not the main cause of ACEs [9]. 

In 1989, Muller and his colleagues used the word “vulnerable” to describe rupture-prone plaques, with characteristics of a large lipid pool, a thin cap, and macrophage-dense inflammation on or beneath its surface [10], as the underlying cause of most clinical coronary events. More and more studies suggested that ACEs were triggered by thrombosis associated with rupture of vulnerable atherosclerotic plaques [11]. The change of plaque from its stable state to an unstable one was not related to the plaque size, quantity, or position or the severity of stenosis. Although PCI improves significant stenosis, it cannot influence the biological course of vulnerable plaque, thus the problem of “unstable” plaque is still unresolved.

In recent years, many clinical trials showed that statins could reduce ACEs significantly yet only improve the luminal size slightly [12]. Experimental researches have proved that statins have potential effects on stability of AS plaques [13]. Stenting (including drug-eluting stents) reduces restenosis and repeated intervention, but does not reduce mortality or myocardial infarction [14]. Therefore, it is necessary for us to reevaluate the benefits of active medicinal treatment and invasive PCI treatment in chronic myocardial ischemia. Based on in-depth understanding of AS pathogenesis, the vascular pathophysiological research has turned to new direction of stabilizing vulnerable plaque and inhibiting thrombosis after plaques rupture. The secondary prevention of CHD also focused on intervention of vulnerable plaque instead of treating luminal stenosis of coronary artery [15, 16].

### 2.2. From Predominant Theory of “Lipid Deposit” to General Acknowledgment of “Inflammatory Reaction” Theory

“Lipids deposit” theory of AS has been put forward for over 100 years based on the causal relation between hyperlipidemia and AS [17]. This theory holds that lipids deposition on the artery wall leads to the AS plaques and has played a very important role in AS pathogenesis for a long period.

In recent years, some researches indicated that AS had the basic manifestation of inflammation: degeneration, exudation, and proliferation. The cell-cell interaction is similar to other chronic inflammation diseases such as rheumatoid arthritis, chronic pancreatitis, and hepatic cirrhosis. With continuous detection of inflammatory cells and mediators, AS was no longer regarded as a simple disease of lipid deposition on vessel wall but also an advancing inflammatory reaction. Recent advances in basic science have established a fundamental role for inflammation in mediating all stages of this diseases from initiation through progression and, ultimately, the thrombotic complications of AS.

In 1999, based on his famous “injury reaction” theory, Ross declared that AS is one of the inflammatory disease [18]. AS is a process of active inflammatory reaction inside the vessel wall rather than a process of passive lipid deposit onto the vessel wall. This theory initiates a new epoch of AS treatment and it leads to deep understanding of cardiovascular diseases: inflammation fuels the development and progression of atherosclerosis as well as causes certain plaques to rupture and subsequent thrombosis, leading to such atherosclerotic complications as heart attack and stroke. High-sensitivity C-reactive protein (hs-CRP) and other blood inflammatory markers may be useful in the estimation of prognosis, risk level in AS patients, and even be a potential target of AS treatment and prevention [19]. Despite regulating blood lipids metabolism, statins should be recommended for their anti-inflammation and other protective effects on cardiovascular diseases. Aspirin can not only inhibit platelet aggregation but also prevent the malfunction of endothelial cells through its anti-inflammation effects [16]. Anti-inflammation has been one of the most important issues of AS research and several strategies that intervene with inflammation reaction are under study.

### 2.3. New Concept from “Vulnerable Plaque” to “Vulnerable Patient”

Plaque rupture is the most common type of plaque complication, accounting for nearly 70% of fatal acute myocardial infarctions and/or sudden coronary deaths. Vulnerable plaque is the main, but not the unique, cause for ACEs. The position of plaque rupture, the size and amount of plaques, coronary spasm, hypercoagulable state, collateral circulation, and the degree of myocardial damage should also be considered. In 2003, an article named “From vulnerable plaque to vulnerable patient: a call for new definitions and risk assessment strategies” was published on *Circulation* written by over fifty of the most famous cardiovascular experts of the world [20, 21]. The new concept of “vulnerable plaque” to “vulnerable patients” has led in a new direction to the prevention of ACEs.

The term “vulnerable patient” is proposed to define subjects susceptible to an acute coronary syndrome or sudden cardiac death based on plaque, blood, or myocardial vulnerability (1-year risk ≥5%). Extensive efforts are needed to quantify an individual's risk of an event according to each component of vulnerability (plaque, blood, and myocardium). Such a comprehensive risk-stratification tool capable of predicting acute coronary syndromes as well as sudden cardiac death would be very useful for preventive cardiology. The new concept of “vulnerable plaque” to “vulnerable patients” stresses evaluating patients as a whole and thus further optimizes overall assessment of cardiovascular risks, and prevents ACEs by early intervention of vulnerable patients.

## 3. An Integrative East-West Medicine Perspective for Future AS Management

The transitions in understanding AS, from local plaques to entire coronary tree and patient as a whole, from passive lipid deposit process to an active inflammatory reaction and cell interaction process, innovate strategies of prevention and treatment for AS and CHD from coronary stenosis-targeted invasive PCI treatment to vulnerable patient-targeted comprehensive assessment, early-detection and preventive medication strategies, happen to mirror “holism concept” “living in harmony with the environment” “preventive treatment of disease” and “treatment based on syndrome differentiation or pattern diagnosis” advocated by TCM. They can also help us fully understand the two different medical systems, Western medicine (WM) and TCM, as well as make the best of the advantages of both of them.

The previous researches have shown that Chinese medicines of activating blood circulation (ABC) could treat AS by multiple ways such as lowering blood lipid, inhibiting platelet adhesion and aggregation, and improving blood viscosity and inhibiting SMC proliferation. In 2003, based on AS models of ApoE-deficient mice, we studied the effects of six ABC herbs (Radix Salviae Miltiorrhizae, Radix Paeoniae Rubra, Rhizoma Chuanxiong, Radix Notoginseng, Semen Persicae, Wine steamed Radix, and Rhizoma Rhei) and a compound preparation (consisting of Chuanxingol and Paeoniflorin) on stabilizing AS plaque and their potential mechanisms. The results indicated that most ABC herbs showed multiple effects on different links of AS, such as regulating blood lipids, influencing collagen metabolism, and anti-inflammatory reaction, thus had potential effect on stabilizing AS plaque [22, 23]. Although the final effect of ABC herbs on stabilizing plaque was slightly less than that of simvastatin, they showed better effects on certain links such as increasing high-density lipoprotein cholesterol (HDL-C), which exhibited the superiorities of Chinese medicine in overall regulation by influencing multiple targets [8]. The superior effect of the compound preparation to either herbal extractive component [24] indicated the synergetic effect based on TCM compatibility theory. Therefore, Chinese herbal medicines, especially compound prescriptions, warrant further investigation and might be an complementary or alternative therapy to statins in stabilizing vulnerable plaque through a synergistic and multitargeted effect.

The new concept of “vulnerable patient” also provides TCM with new opportunity in detecting high-risk CHD patients and further reducing ACEs by early intervention. Under the guidance of TCM holism concept and thought of treatment based on syndrome differentiation, we conducted a multicenter cohort study, enrolling stable CHD patients and documenting one-year follow-up cardiovascular endpoint events. Prognosis-related factors, including past medical history, symptoms, body signs, biochemical indicators, and tongue manifestations, were identified to establish an integrative risk-assessment system for detecting high-risk CHD patients [25–27]. A large-scale randomized controlled trial aiming at early intervening high-risk CHD patients based on this integrative risk assessment system is about to start soon. Given the convergence of both the East and the West conceptualization of AS, it is hopeful that this integrative East-West strategy will facilitate early detection and more effective treatment for the vulnerable patients with CHD and other AS-related diseases.
